# Gravity-assisted opacification method for confirming hilar biliary obstruction

**DOI:** 10.1055/a-2253-1069

**Published:** 2024-02-15

**Authors:** Yuzo Shimokawa, Hirotada Akiho, Nao Fujimori

**Affiliations:** 1 37060 Department of Gastroenterology, Kitakyushu Municipal Medical Center, Kitakyushu, Japan; 2 Department of Medicine and Bioregulatory Science, Kyushu University Graduate School of Medical Sciences, Fukuoka, Japan

Regardless of various strategies for draining malignant hilar obstructions 1 2 , clear guidelines on which biliary branches should be drained are lacking. Branches that are not drained and opacified are at high risk for segmental cholangitis and a poor prognosis 3 . We propose a novel, simple method based on the principle “heavier objects tend to flow downward” for identifying branches to be drained by leveraging the gravitational movement of the contrast medium.

The three major branches should be monitored: the posterior branch, anterior branch, and left hepatic duct. In the prone position, which is common during endoscopic retrograde cholangiopancreatography, the posterior branch is usually located at the highest point and the left hepatic duct at the lowest point ( Fig. 1 **a–d** ).

**Fig. 1:**
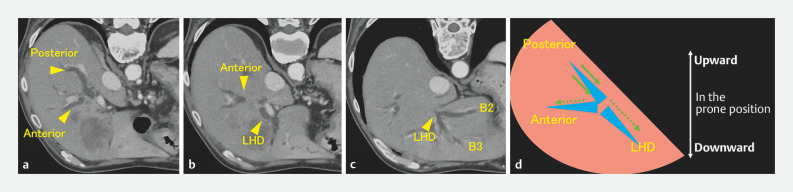
Computed tomography images and the corresponding schematic figures illustrate the vertical positions of the biliary branches in the prone position. **a** The posterior branch extends backward. **b** The anterior branch is situated between the posterior branch and the left hepatic duct. **c** The left hepatic duct is present ventral to the other two major branches. **d** The contrast medium injected into the posterior branch moves centrally (solid arrow). If it is unobstructed, it also moves to the anterior branch or left hepatic duct (dashed arrow).

By initially injecting contrast medium into a branch presumed to be the posterior branch, we were able to verify its identity and assess whether it was occluded by the other two branches. If the contrast medium remains on the central side due to gravity, this indicates that the branch is likely the posterior branch as it extends backward (upward in the prone position) ( Fig. 2 **a–d** ). Similarly, if the contrast medium does not flow from the posterior branch to the other branches, an obstruction requiring drainage is determined ( Fig. 2 **e–h** ).

**Fig. 2:**
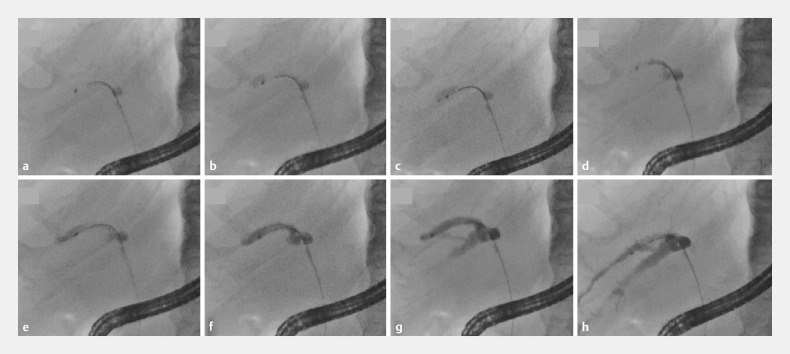
Fluorographic images depict the identification of the posterior branch and confirmation of its obstruction. **a** An endoscopic retrograde cholangiopancreatography (ERCP) catheter is advanced into a biliary branch, presumed to be the posterior branch. **b** Injection of the contrast medium commenced. **c** Injection of the contrast medium is halted shortly. **d** Injected contrast medium has gravitated centrally. **e** Injection of the contrast medium has resumed. **f** The posterior branch is being filled with the contrast medium. **g** As the contrast medium is injected, it moves to the ventral area within the posterior branch. **h** Despite near-complete opacification of the posterior branch, the other two major lower branches remain unopacified, indicating obstruction in the posterior branch.

We present a case of hilar cholangiocarcinoma ( Video 1 ). Obstruction in the posterior branch requiring drainage was confirmed because the injected contrast medium did not reach the other branches ( Fig. 3 ). Metallic stents were deployed in all three branches in a stent-in-stent manner, and good drainage was confirmed by aspiration and reopacification from the distal bile duct ( Fig. 4 **a–d** ).

Gravity-assisted opacification method for confirming hilar biliary obstruction.Video 1

**Fig. 3:**
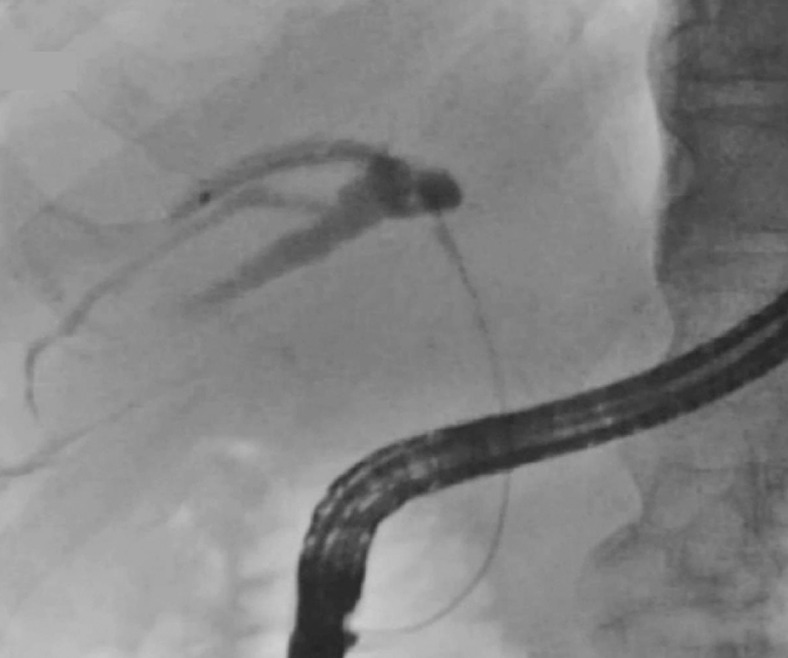
Fluorographic image. Initial opacification of the posterior branch without duplication confirmed its obstruction.

**Fig. 4:**
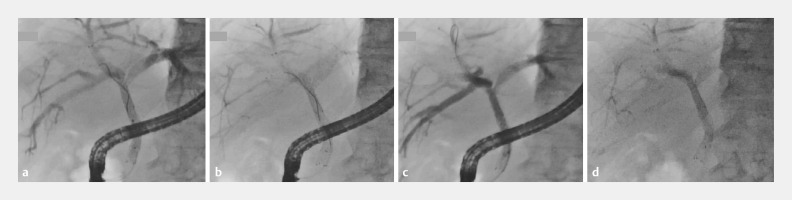
Fluorographic images confirm the patency of the deployed stents. **a** The contrast medium remains in the branches immediately after stent deployment. **b** The contrast medium is effectively aspirated through the deployed stents from the common bile duct. **c** The contrast medium is re-injected from the common bile duct, resulting in good opacification of all three major branches. **d** By the end of the ERCP procedure, almost all contrast medium had drained effectively.

In conclusion, initial opacification of a branch that extends backward (upward in the prone position) can indicate obstruction by other branches. This simple opacification method, leveraging gravitational force, can assist in precise biliary tree evaluation and segmental cholangitis prevention.

Endoscopy_UCTN_Code_TTT_1AR_2AB

## Notes

Endoscopy E-Videos https://eref.thieme.de/e-videos
*E-Videos* is an open access online section of the journal *Endoscopy* , reporting on interesting cases and new techniques in gastroenterological endoscopy. All papers include a high-quality video and are published with a Creative Commons CC-BY license. Endoscopy E-Videos qualify for HINARI discounts and waivers and eligibility is automatically checked during the submission process. We grant 100% waivers to articles whose corresponding authors are based in Group A countries and 50% waivers to those who are based in Group B countries as classified by Research4Life (see: https://www.research4life.org/access/eligibility/ ). This section has its own submission website at https://mc.manuscriptcentral.com/e-videos .
